# Nasal Screening for MRSA: Different Swabs – Different Results!

**DOI:** 10.1371/journal.pone.0111627

**Published:** 2014-10-29

**Authors:** Philipp Warnke, Hagen Frickmann, Peter Ottl, Andreas Podbielski

**Affiliations:** 1 Institute of Medical Microbiology, Virology, and Hygiene, Rostock University Hospital, Rostock, Germany; 2 Department of Tropical Medicine at the Bernhard Nocht Institute, German Armed Forces Hospital Hamburg, Hamburg, Germany; 3 Department of Prosthodontics and Material Sciences, Rostock University Hospital, Rostock, Germany; Universitätsklinikum Hamburg-Eppendorf, Germany

## Abstract

**Objectives:**

Swab-based nasal screening is commonly used to identify asymptomatic carriage of *Staphylococcus aureus* in patients. Bacterial detection depends on the uptake and release capacities of the swabs and on the swabbing technique itself. This study investigates the performance of different swab-types in nasal MRSA-screening by utilizing a unique artificial nose model to provide realistic and standardized screening conditions.

**Methods:**

An anatomically correct artificial nose model was inoculated with a numerically defined mixture of MRSA and *Staphylococcus epidermidis* bacteria at quantities of 4×10^2^ and 8×10^2^ colony forming units (CFU), respectively. Five swab-types were tested following a strict protocol. Bacterial recovery was measured for direct plating and after elution into Amies medium by standard viable count techniques.

**Results:**

Mean recovered bacteria quantities varied between 209 and 0 CFU for MRSA, and 365 and 0 CFU for *S. epidermidis*, resulting swab-type-dependent MRSA-screening-sensitivities ranged between 0 and 100%. Swabs with nylon flocked tips or cellular foam tips performed significantly better compared to conventional rayon swabs referring to the recovered bacterial yield (p<0.001). Best results were obtained by using a flocked swab in combination with Amies preservation medium. Within the range of the utilized bacterial concentrations, recovery ratios for the particular swab-types were independent of the bacterial species.

**Conclusions:**

This study combines a realistic model of a human nose with standardized laboratory conditions to analyze swab-performance in MRSA-screening situations. Therefore, influences by inter-individual anatomical differences as well as diverse colonization densities in patients could be excluded. Recovery rates vary significantly between different swab-types. The choice of the swab has a great impact on the laboratory result. In fact, the swab-type contributes significantly to true positive or false negative detection of nasal MRSA carriage. These findings should be considered when screening a patient.

## Introduction

Nasal carriage of *S. aureus* is present in 20–30% of the population [1], [2] and is a major risk factor for various purulent endogenous infections as well as bacterial transmission both in community and nosocomial environments [2], [3], [4]. Methicillin-resistant *Staphylococcus aureus* (MRSA) are the most frequent cause for complicated nosocomial infections [5], [6], [7], [8], [9], [10], [11]. As *S. aureus* predominantly colonizes the anterior part of the nasal cavity [12] swab based screening techniques are commonly used to identify such carriers.

Combinations of different swabs and transport systems have been evaluated for in vitro and in vivo performance data in the past with a broad variation of results concerning the bacterial yield [13], [14], [15], [16], [17], [18], [19], [20]. Additionally, utilizing the correct swabbing technique can significantly improve the bacterial recovery rate in nasal screening even within the same swab-type [21]. Furthermore, commonly used swabs with different tips, like rayon, cellular foam, or nylon flocked tips, vary significantly with respect to uptake and release of liquid and bacteria, depending on the clinical setting, i.e. if used on dry surfaces such as skin and other epithelia or on wet surfaces such as operation wounds [22].

There are recommendations of the Clinical and Laboratory Standards Institute (CLSI) how to perform quality controls for microbiological transport systems proposing that the inoculation volume is pipetted into tubes or wells of a microtiter plate and the swab is placed into the tube or well [23], but these recommendations do not consider inter-individual differences in patients. Yet, clinical swab studies have to deal with these inter-individual differences concerning anatomy, surface-moisture, or bacterial densities in patients' noses and therefore can hardly be standardized. To address these issues this study followed a new approach by utilizing a recently introduced, anatomically correct, artificial nose model [21], [24] inoculated with a defined mixture of MRSA and *S. epidermidis* to analyze bacterial recovery rates from different swab-types by direct plating and after elution into Amies medium, respectively. Thus, anatomical and mechanical challenges as well as a high degree of laboratory reproducibility are combined in a nasal MRSA-screening study for the first time.

## Materials and Methods

### Swabs

The following swabs were tested:

1. MWE medical wire, Corsham Wiltshire England, Tubed Sterile Dryswab, rayon, ref. MW102;
2. MWE medical wire, Corsham Wiltshire England, Sigma Dry Swab Tubed, Σ-Swab, polyurethane cellular foam, ref. MW941;
3. Mast Group Ltd., Reinfeld, Germany, MASTASWAB MD 555, rayon, via Copan, Brescia, Italy, ref. 800155;
4. Copan, Brescia, Italy, FLOQSwabs, eSwab, ref. 490CE.A. Sterile single use sample collection pack containing:
  pink polypropylene screw-cap tube with internal conical shape filled with 1 ml of liquid Amies medium
  one regular size applicator swab with flocked nylon fiber tip.
  Swabs from the set were used either separately or in combination with provided liquid Amies preservation medium;
5. Sarstedt, Nuembrecht, Germany, neutral swab, rayon, via Copan, Brescia, Italy, cat.no. 80.1301.

For elution experiments Amies medium from Copan sample collection pack (see swab no. 4, Copan ref. 490CE.A) was used for all swab-types.

### Bacterial culture techniques

*Staphylococcus aureus* MRSA strain (ST22-MRSA-IV, Barnim epidemic strain) and *Staphylococcus epidermidis* strain (DSMZ 1798) were separately propagated at 37°C in brain-heart-infusion (BHI) medium as overnight standing cultures in ambient air. Early stationary phase cells were harvested, washed in phosphate-buffered saline (PBS; NaCl (137 mmol/l), KCl (2.7 mmol/l), Na_2_HPO_4_×2 H_2_O (10 mmol/l), KH_2_PO_4_ (2.0 mmol/l)) at pH 7.4 and resuspended in BHI medium+20% glycerol. Aliquots were stored at −80°C for up to 3 months. After 3 d conservation in the freezer, stock concentration was determined by viable cell count of 3 tubes. Therefore, tube content was transferred into 1000 µl PBS (8000 rpm, 4°C, 5 min (5417R Eppendorf)) followed by 1∶10 serial dilution steps in PBS and cultivation of 100 µl aliquots on Columbia agar supplemented with 5% sheep blood at 37°C under ambient atmosphere for 48 h.

### Inoculation of the nose models

Nose models (Figure S1) were prepared for each test series at the day of usage. Autoclaved, sterile nose models were inoculated with a suspension of *Staphylococcus aureus* ST22-MRSA-IV and *Staphylococcus epidermidis* DSMZ 1798 bacterial strains at quantities of 4×10^2^ and 8×10^2^ CFU, respectively. Four 10 µl droplets of bacterial suspension were applied within the nasal vestibules. The nose models were then dried for one hour at room temperature.

### Swabbing technique

Nose models were swabbed according to Warnke et al. [21]. Each nasal vestibule was swabbed by circulating five times while rotating the swab and exerting gentle pressure.

### Detection of bacteria

All swabs were placed into the corresponding transport tube and were subjected to microbiological analysis one hour after swabbing the nose models to simulate optimum transport conditions. CFU were determined by streaking swabs in a standardized fashion onto Columbia agar supplemented with 5% sheep blood in 5 streaks with a length of 5 cm while constantly rotating the swab shaft in an angle of 45° to the plate and exerting gentle pressure (swab shaft was slightly bended).

For the elution of Copan FLOQSwabs according to manufacturer's instructions, the swabs were rotated (10 turns) in 1 ml eSwab liquid Amies preservation medium, Copan, Brescia, Italy, ref. 490CE.A. 100 µl aliquots were plated onto Columbia agar supplemented with 5% sheep blood.

Agar plates were subsequently cultured at 37°C under ambient atmosphere for 48 h.

CFU were then counted by macroscopic inspection. *Staphylococcus aureus* was distinguished from *Staphylococcus epidermidis* by hemolysis (β-hemolysis vs. no hemolysis) and colony color (golden yellow vs. white), if necessary by agglutination assay (Slidex Staph Plus, bioMérieux, Marcy l'Etoile, France).

### Positive control

For each test series quantities of inoculated bacteria were controlled by directly plating serial dilutions of the bacterial suspension onto Columbia agar supplemented with 5% sheep blood followed by cultivation at 37°C under ambient atmosphere for 48 h and CFU counting. For analysis of bacterial recovery rates, positive controls were defined as 100%.

### Negative control

In each test series, autoclaved, non-inoculated nose models were swabbed by one swab of each swab-type. Swabs were plated on Columbia agar supplemented with 5% sheep blood followed by culture at 37°C under ambient atmosphere for 48 h. Results from corresponding experiments were only accepted if no bacteria could be detected by this procedure.

### Iteration of experiments

All experiments were performed in quintuplicate (technical replicates) and repeated on three independent time points (biological replicates).

### Statistical analysis

Data were analyzed using nonparametric Wilcoxon-Mann-Whitney U-test. All p values resulted from two-tailed statistical test. p-values of <0.05, <0.01, and <0.001 were considered to be marginally significant, significant, and highly significant, respectively.

### Ethics statement

The study was performed without using human or animal subjects and/or tissues.

## Results

### Quantitative recovery of bacteria

**Figure 1:**
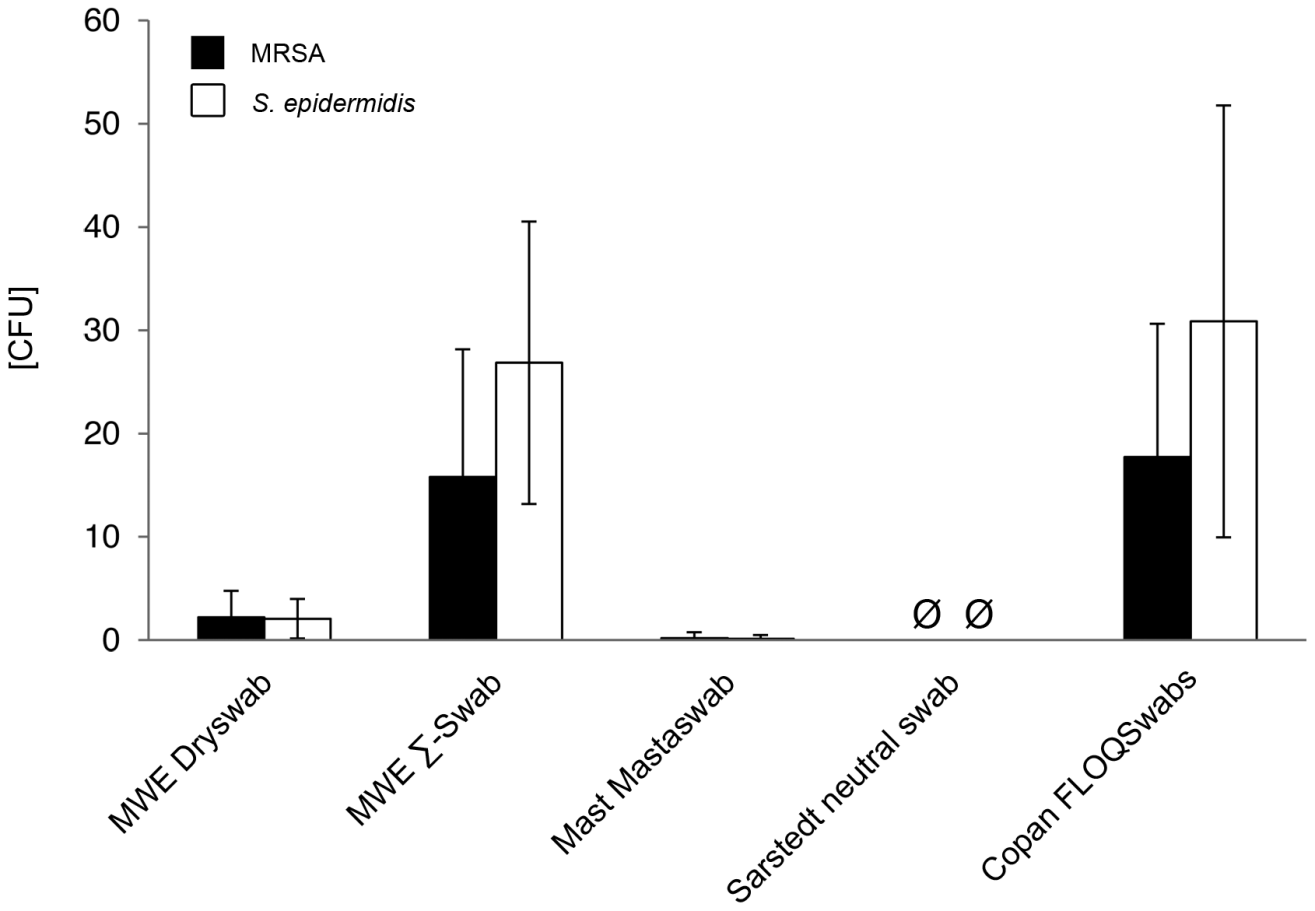
Recovery of bacteria in absolute numbers after direct plating. Viable counts of bacteria by direct plating of the displayed swab-types were determined by CFU counting as described in the methods section. CFU = colony forming units. Results from statistical analysis are shown in Table S1.

**Figure 2:**
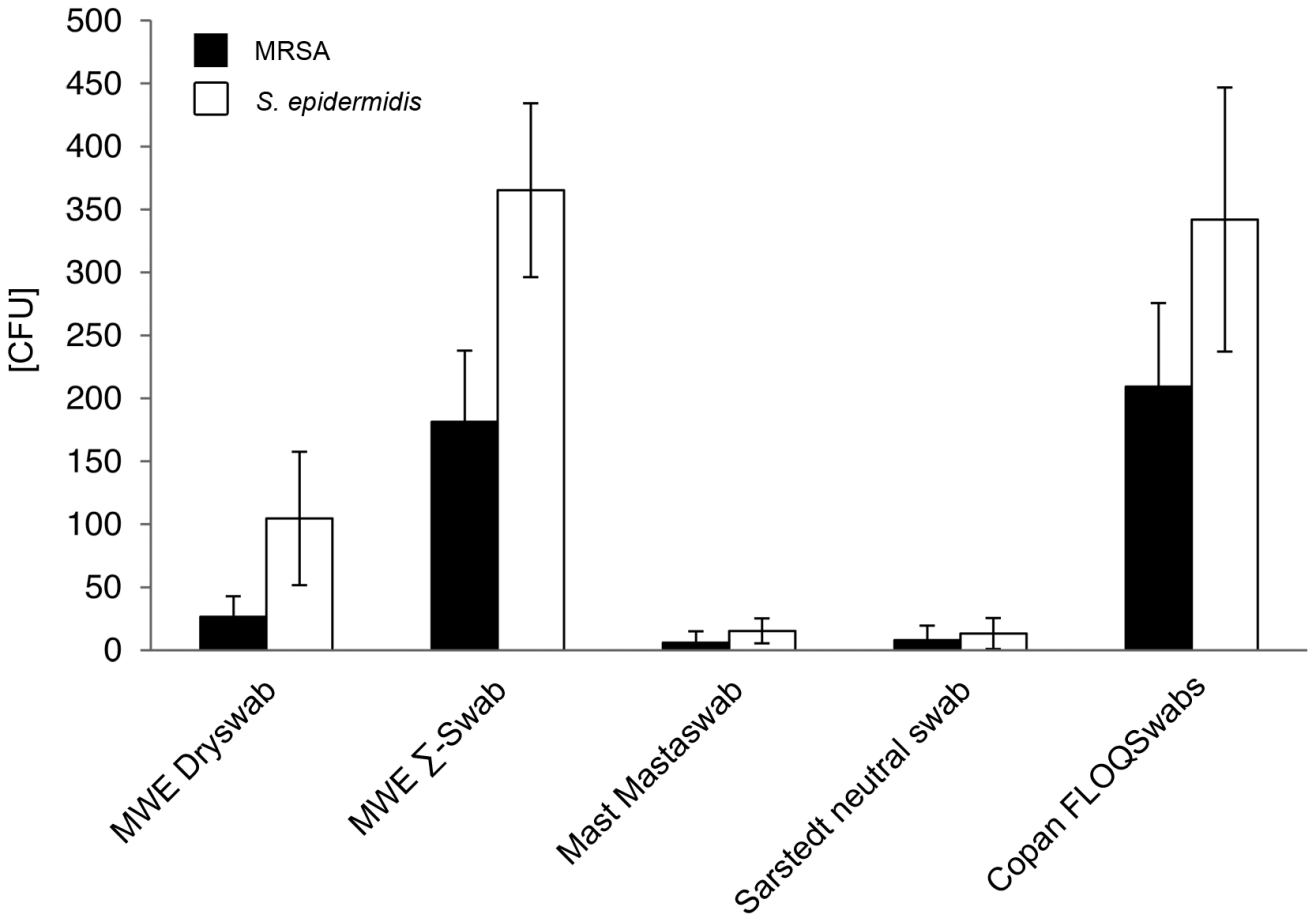
Recovery of bacteria in absolute numbers after elution into Amies medium. Viable counts of bacteria after elution of the displayed swab-types into Amies medium were determined by CFU counting as described in the methods section. CFU = colony forming units. Results from statistical analysis are shown in Table S1.

Quantitative recovery of bacteria varied highly significant between 209 and 0 CFU for MRSA and 365 and 0 CFU for *S. epidermidis* (p<0.001). After direct plating, highest bacterial amounts for both bacterial species were obtained by utilizing Copan FLOQSwabs and MWE Σ-Swab. Compared to MWE Dryswab, Mast Mastaswab, and Sarstedt neutral swab, the differences were highly significant (p<0.001). Both swab types, i.e. Copan FLOQSwabs and MWE Σ-Swab, performed equally well, with no significant difference for MRSA- (p = 0.744) as well as *S. epidermidis-* (p = 0.837) detection. MWE Dryswab and Mast Mastaswab performed poorly with CFU amounts close to the detection limit. When utilizing Sarstedt neutral swab no bacteria could be recovered at all (Figure 1, Table S1). After elution into Amies medium, again, highest bacterial amounts were obtained by Copan FLOQSwabs and MWE Σ-Swab, with no significant difference between those two swab-types for MRSA- (p = 0.325) as well as *S. epidermidis-* (p = 0.539) detection. Compared to MWE Dryswab, Mast Mastaswab, and Sarstedt neutral swab, the differences were highly significant (p<0.001) (Figure 2, Table S1). Comparing differences within the same swab-type with or without elution into Amies medium, MRSA recovery after elution into Amies medium compared to direct plating could be significantly increased for MWE Dryswab, MWE Σ-Swab, and Copan FLOQSwabs, while elution had no impact on CFU recovery when using Mast Mastaswab (p = 0.137) and only marginal impact on the CFU recovery when using Sarstedt neutral swab (p<0.05) (Table S1).

In particular, for direct plating mean bacterial recovery counts were measured as follows: for MRSA: MWE Dryswab 2.2 CFU, MWE Σ-Swab 15.8 CFU, Mast Mastaswab 0.2 CFU, Sarstedt neutral swab 0.0 CFU, Copan FLOQSwabs 17.7 CFU; for *S. epidermidis*: MWE Dryswab 2.1 CFU, MWE Σ-Swab 26.9 CFU, Mast Mastaswab 0.1 CFU, Sarstedt neutral swab 0.0 CFU, Copan FLOQSwabs 30.9 CFU (Figure 1, Table S3).

Since for Mast Mastaswab and Sarstedt neutral swab little or no CFU could be detected, the sensitivity thresholds to achieve positive results for these swab-types were additionally determined. For MRSA/*S. epidermidis* suspensions, detection limits for two swab-types were approximately at 7–8×10^2^ CFU and 1.4–1.6×10^3^ CFU, respectively (Table S4).

After elution into Amies medium, mean bacterial recovery counts were measured as follows: for MRSA: MWE Dryswab 26.7 CFU, MWE Σ-Swab 181.3 CFU, Mast Mastaswab 6.0 CFU, Sarstedt neutral swab 8.0 CFU, Copan FLOQSwabs 209.3 CFU; for *S. epidermidis*: MWE Dryswab 104.7 CFU, MWE Σ-Swab 365.3 CFU, Mast Mastaswab 15.3 CFU, Sarstedt neutral swab 13.3 CFU, Copan FLOQSwabs 342.0 CFU (Figure 2, Table S3).

### Relative recovery of bacteria

**Figure 3:**
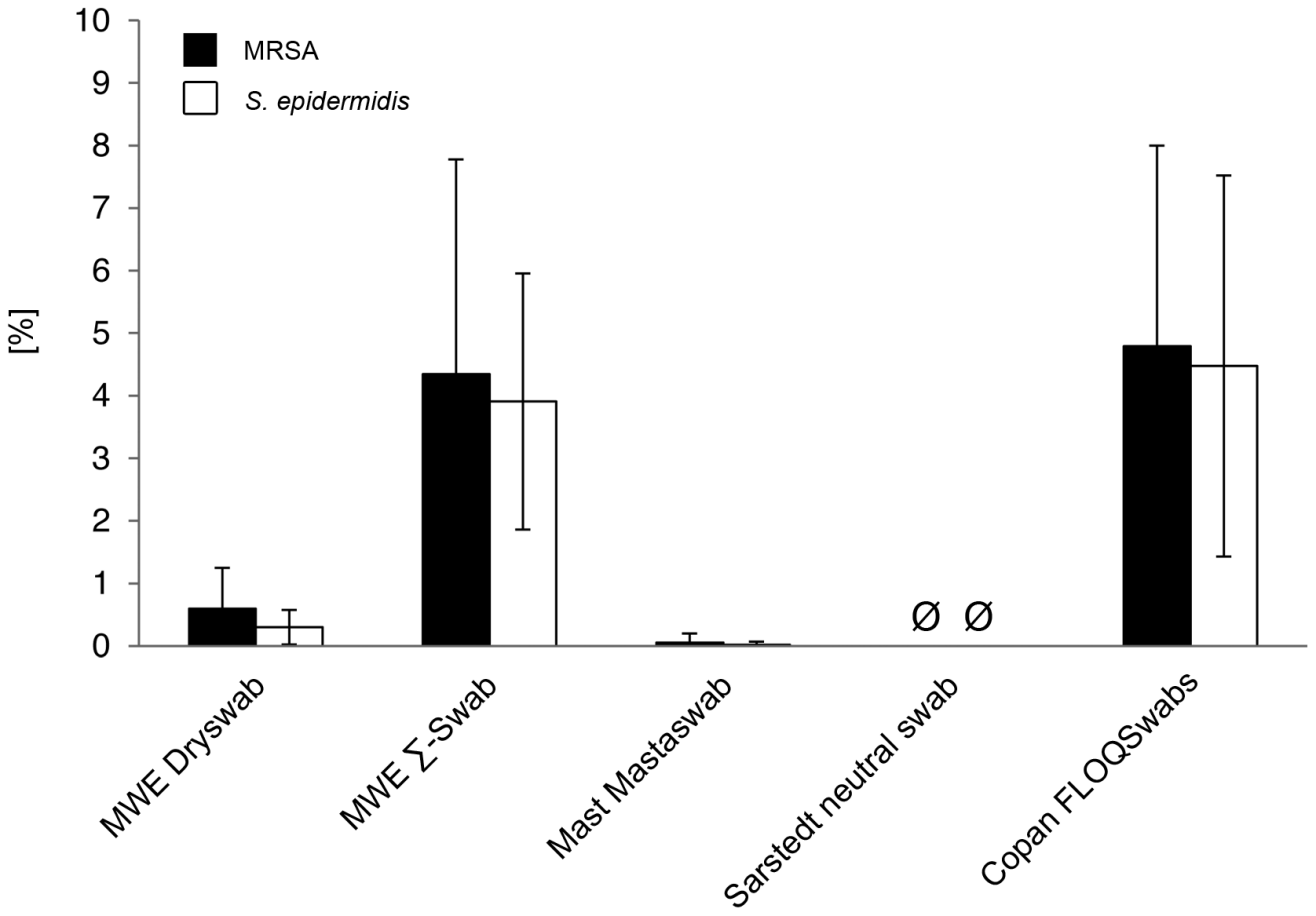
Relative recovery of bacteria compared to inoculation dose after direct plating. Ratios were determined by comparison of viable counts of released bacteria to the inoculation dose, which was defined as 100%. Results from statistical analysis are shown in Table S2.

**Figure 4:**
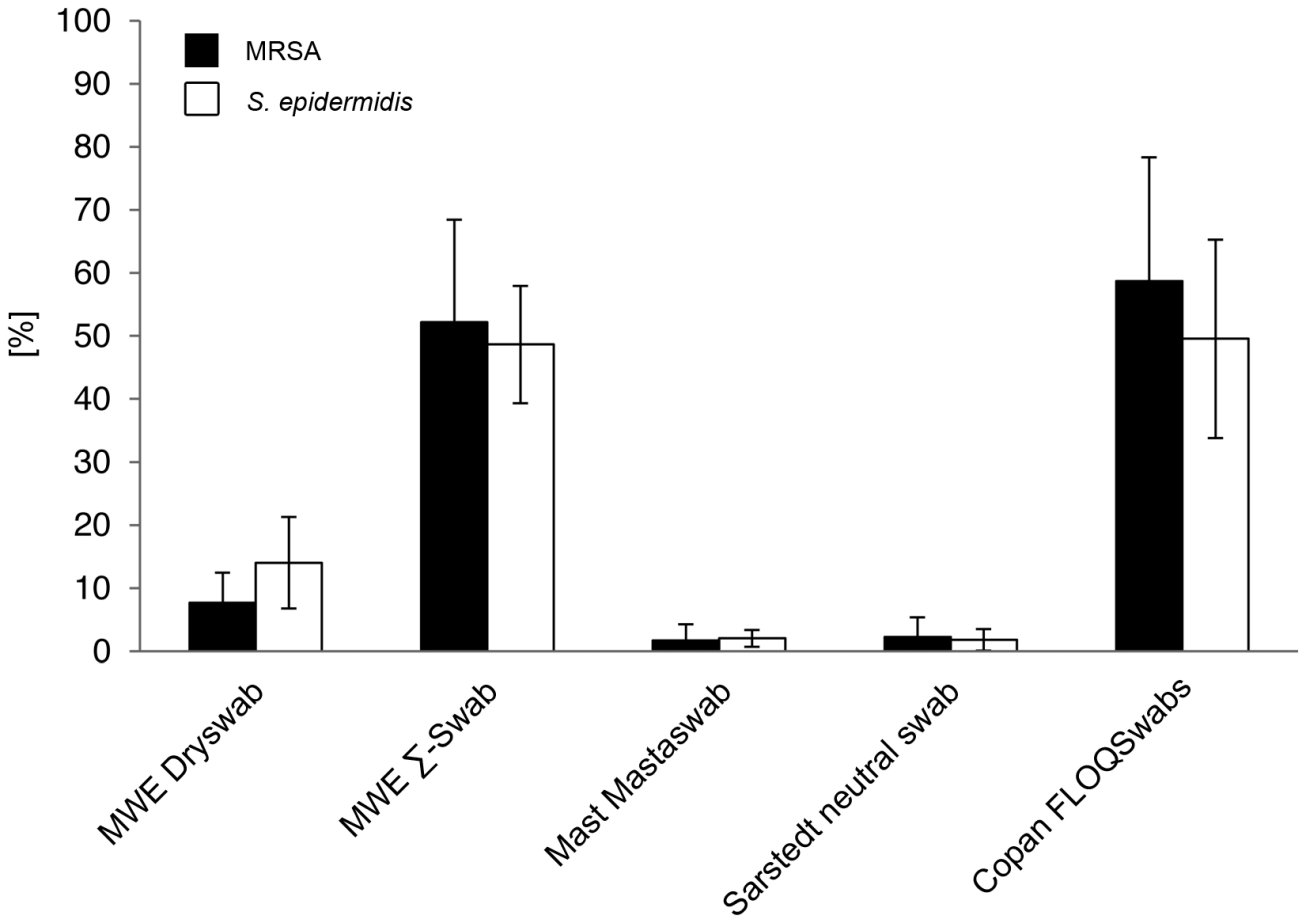
Relative recovery of bacteria compared to inoculation dose after elution into Amies medium. Ratios were determined by comparison of viable counts of released bacteria to the inoculation dose, which was defined as 100%. Results from statistical analysis are shown in Table S2.

The amount of the recovered bacteria was compared to the inoculation dose, which was stated as 100% in each individual experiment. Mean relative recovery rates were measured by direct plating as follows: for MRSA: MWE Dryswab 0.6%, MWE Σ-Swab 4.3%, Mast Mastaswab 0.1%, Sarstedt neutral swab 0.0%, Copan FLOQSwabs 4.8%; for *S. epidermidis*: MWE Dryswab 0.3%, MWE Σ-Swab 3.9%, Mast Mastaswab 0.0%, Sarstedt neutral swab 0.0%, Copan FLOQSwabs 4.5% (Figure 3). Mean relative recovery rates after elution into Amies medium were measured as follows: for MRSA: MWE Dryswab 7.7%, MWE Σ-Swab 52.2%, Mast Mastaswab 1.7%, Sarstedt neutral swab 2.3%, Copan FLOQSwabs 58.7%; for *S. epidermidis*: MWE Dryswab 14.0%, MWE Σ-Swab 48.7%, Mast Mastaswab 2.0%, Sarstedt neutral swab 1.8%, Copan FLOQSwabs 49.6% (Figure 4). Since statistical analysis was performed on absolute CFU counts (Figure 1, Table S1), it was not repeated on the relative ratios.

To analyze species and concentration dependent differences, recovery of MRSA (in percent) was compared to *S. epidermidis* within each swab-type. Within the range of the utilized bacterial concentrations, recovery ratios of the particular swab-types were independent of the bacterial species after direct plating as well as after elution into Amies medium (p>0.05), with marginally significant differences for MWE Dryswab after elution into Amies medium (p<0.05) (Table S2).

### Qualitative MRSA detection

Inoculated nose models were stated as 100% MRSA positive. Sensitivity in qualitative MRSA detection was analyzed for each swab-type by rating a recovery of >1 CFU MRSA as a positive result, revealing sensitivities between 0 and 100%. By direct plating, Copan FLOQSwabs performed best with 100% sensitivity, followed by MWE Σ-Swab and MWE Dryswab with 93% and 73% sensitivity, respectively. Mast Mastaswab and Sarstedt neutral swab achieved sensitivities of 13% and 0%, respectively (Table 1). After Elution into Amies medium, sensitivities ranged between 40 and 100%. In particular, MWE Σ-Swab and Copan FLOQSwabs achieved a sensitivity of 100%, followed by MWE Dryswab with 93%, Sarstedt neutral swab with 47% and Mast Mastaswab with 40% sensitivity (Table 1).

**Table 1:** Qualitative MRSA detection.

	Sensitivity (%)
MWE Dryswab	11/15 (73)
MWE Σ-Swab	14/15 (93)
Mast Mastaswab	2/15 (13)
Sarstedt neutral swab	0/15 (0)
Copan FLOQSwabs	15/15 (100)
MWE Dryswab in Amies medium	14/15 (93)
MWE Σ-Swab in Amies medium	15/15 (100)
Mast Mastaswab in Amies medium	6/15 (40)
Sarstedt neutral swab in Amies medium	7/15 (47)
Copan FLOQSwabs in Amies medium	15/15 (100)

Legend: Artificial nose models were inoculated with bacterial suspensions of MRSA and *S. epidermidis* and defined as 100% MRSA positive. After swabbing with five swab-types (*n* = 15 swabs per type) MRSA detection rates were analyzed by direct plating or after elution of swab contents into Amies medium. Detection of >1 CFU MRSA on Columbia agar plates supplemented with 5% sheep blood was rated as a positive result. Results of sensitivity analysis are displayed as followed: number of positive results/number of maximal positive results (percentage).

## Discussion

Swab-based nasal screening is the most common technique to detect nasal carriage of MRSA in patients. As swab tips vary by size and material, it is obvious that these parameters individually influence the process of specimen collection. Therefore, testing of different swab-types within the intended clinical setting of usage is important to identify those swabs, which perform best referring to bacterial recovery rates and test sensitivities.

The Clinical Laboratory Standards Institute (CLSI) published recommendations for swab-testing with standardized laboratory conditions [23]. The recommended methods do not address anatomical and mechanical challenges, which do exist within patients. For testing according to the CLSI, swabs are placed into vials and exposed to predefined amounts of bacterial suspensions. This procedure ensures reproducibility of test results, but does not necessarily reflect the clinical situation. Although studies on patients reflect the clinical situation, they do not provide standardized conditions with respect to anatomy or bacterial densities.

To address both points, the present study utilizes an artificial, anatomically correct model of a human nose [21], [24] inoculated with numerically defined quantities of MRSA and *S. epidermidis*. For *S. aureus*, ST22-MRSA-IV strain, i.e. the Barnim epidemic strain, was chosen, since it is the predominant MRSA-strain in many countries [25], [26], [27], [28], [29], [30], [31], [32], [33]. *S. epidermidis* was chosen because of its role as a physiological component of nose cavity microflora worldwide and consecutively, as a typical contaminant in nasal swab samples [34], [35], [36], [37], [38], [39], [40]. A ratio of 2∶1 (*S. epidermidis*: MRSA) was selected to reflect the dominance of the physiological human nasal microflora and to analyze swab-performance under influence of potential bacterial interference.

To achieve optimal bacterial recovery rates, nasal swabbing followed a recently published strict protocol. In that study it could be demonstrated, that the correct swabbing technique resulted in significantly higher bacterial recovery [21]. Before plating, swabs were stored at room temperature for 1 h to simulate optimal transport time. Longer storage periods were shown to result in substantial loss of target bacteria or overgrowth by contaminants [13], [41], [42], [43], [44].

With respect to bacterial recovery rates in nasal MRSA-screening, the present study revealed significantly better performance of flocked and cellular foam tipped swabs compared to conventional rayon tipped swabs. This data corresponds to previously published studies showing a higher bacterial yield for these swab-types in various situations [22], [42], [45], [46], [47]. Furthermore, in comparison to the use of rayon swabs, flocked swabs revealed an improved uptake of epithelial cells and viruses [48], [49], released more microorganisms in vitro [42], and enhanced the molecular detection of *C. trachomatis* and *N. gonorrhoeae* [50]. The bacterial yield of flocked swabs could be augmented when using Amies preservation medium for transport of swabs [22], [42], [47]. With the present study, this increment could also be demonstrated for MWE Σ-Swab and MWE Dryswab.

In this study the bacterial recovery rates from all tested swab-types were lower than in a previous study, when in a volume-restricted setting identical swab-types were inoculated with bacteria by placing them into bacterial suspensions containing vials [22]. We think that is due to mechanical disturbance or disruption of the swab structure by streaking over a comparatively dry surface. Probst et al. demonstrated that surface exposure of rayon swabs affects the fiber structure which in turn resulted in a trapping of the absorbed bacteria [51]. This goes in line with the findings in the present study, since elution into Amies medium did not or rather marginally improve the bacterial recovery by utilizing Mast Mastaswab and Sarstedt neutral swab.

The convincing in vitro data do not necessarily imply that the flocked and cellular foam swab-types are superior in all clinical situations. It was demonstrated that the bacterial yield from a given swab-type depends on the clinical setting. Compared to flocked or cellular foam swabs, conventional swabs, like rayon swabs, do perform better in settings with unlimited supply of liquid, i.e. surgery wounds [22]. In turn, flocked or cellular foam swabs are superior in case of limited supply of liquid, i.e. screening situations or skin swabs [22]. With the present study these in vitro results could be confirmed as the flocked or cellular foam swabs performed significantly better under the close-to-reality conditions of the utilized nose model. This is probably due to the relatively dry surfaces of the nasal vestibulum, which is reflected by this model.

Of note, sensitivity rates in the present nasal screening study varied swab-type-dependent between 0 and 100% for direct plating, and to various extent could be improved by elution into Amies medium. Consecutively, with some swab-types up to 100% of the screened patients could receive a false negative diagnose of their MRSA carrier status, with all consequences for hygiene management and facilitated spreading of MRSA. Since MRSA transmission is associated with higher healthcare costs [52], [53], [54], [55], [56], [57], [58], [59], [60], [61], [62], such poor test performance could finally affect this important issue.

The inoculum size used in this study was relatively low, but was geared to previous findings yielding mean MRSA nasal colonization densities of 794 CFU [63] and mean *S. aureus* colonization densities of 10 CFU for intermittent and 63 CFU for persistent carriers [64], respectively. Thus, the inoculum size of this study corresponds to MRSA amounts in roughly one third of patients [65]. Therefore, a comparatively poor test sensitivity for some swab-types would be a minor issue for the majority of MRSA carriers, especially since test sensitivity can be boostered by including a broth culture incubation step [66], [67], [68]. However, usage of broth culture prolongs MRSA detection by one day. This in turn could unnecessarily place a non-carrier under more lavish conditions in hospitals with a preemptive isolation concept or an yet undetected carrier under conditions of increased transmission risk. In addition, both broth culture and swabs with poor test sensitivity undermine quantification of MRSA amounts in nasal cavities. Yet, information on bacterial numbers is useful, since a high concentration of *S. aureus* in the nares is a risk factor for subsequent invasive infection [65], [69] and is an independent risk factor for the development of a surgical site infection [70].

As in many hospitals only one swab-type is used for MRSA-screening and infection diagnostics, high test sensitivity will also contribute to a more sensitive and most probably, also faster detection of relevant infectious agents.

## Conclusions

Using a novel, close to real-life conditions approach for swab testing, this study outlines the huge impact of the swab-type on the laboratory results. In fact, the choice of the swab-type could decide on diagnosing a false negative MRSA carrier status. Swabs with nylon flocked tips or cellular foam tips perform much better in nasal MRSA screening than conventional rayon swabs. Swab testing with close to real conditions reveals lower MRSA recovery rates compared to in vitro swab testing according to laboratory standards recommendations. The utilized nose model provides the possibility to test swabs under more realistic conditions - with anatomical and mechanical challenges - and is highly recommended for testing established and new swab-types in nasal screening settings.

## Supporting Information

Figure S1 **Nose model.** Picture of the nose model utilized in this study.
(JPG)

Table S1 **Statistics on quantitative recovery of bacteria.** All p values result from nonparametric, two-tailed Wilcoxon-Mann-Whitney U-test. CFU = colony forming units.
(DOCX)

Table S2 **Statistics on relative recovery of bacteria.** All p values result from nonparametric, two-tailed Wilcoxon-Mann-Whitney U-test.
(DOCX)

Table S3 **Raw data.** Results of CFU counting for each experiment are displayed.
(DOCX)

Table S4 **Detection limits of swabs with low sensitivities.** The minimal bacterial quantities, necessary to achieve positive results after direct plating of the swabs, are displayed for Mast Mastaswab and Sarstedt neutral swab.
(DOCX)
