# Supplementary figures and images for: Nasal Screening for MRSA: Different Swabs – Different Results!
Source: PLoS One. 2014 Oct 29;9(10):e111627. doi: 10.1371/journal.pone.0111627 (PMC4213029; doi:10.1371/journal.pone.0111627)

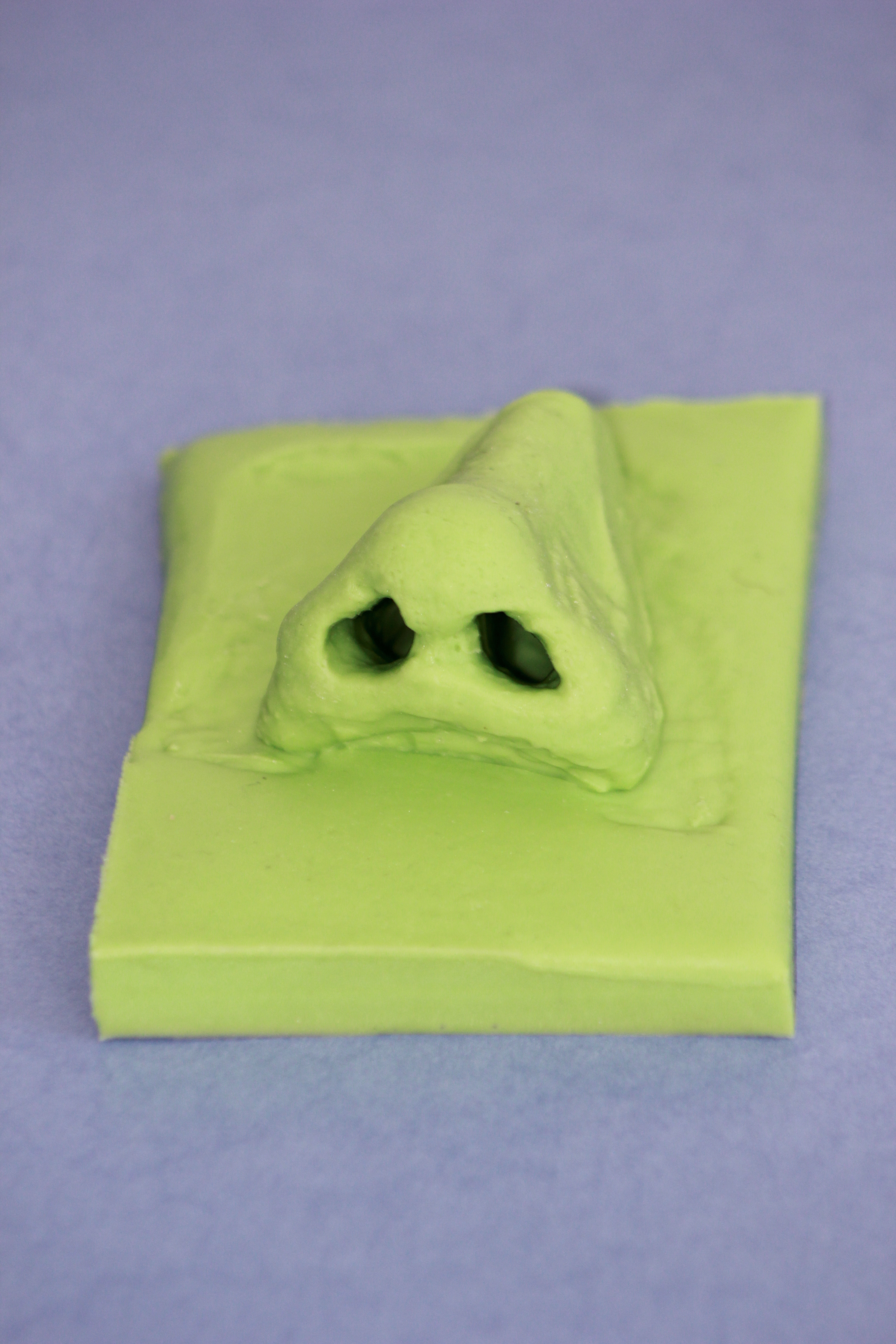

Supplement: Figure S1 — Nose model. Picture of the nose model utilized in this study. (JPG) [file pone.0111627.s001.jpg]
