# Supplementary material for: Nasal Screening for MRSA: Different Swabs – Different Results!
Source: PLoS One. 2014 Oct 29;9(10):e111627. doi: 10.1371/journal.pone.0111627 (PMC4213029; doi:10.1371/journal.pone.0111627)
Supplement: Table S1 — Statistics on quantitative recovery of bacteria. All p values result from nonparametric, two-tailed Wilcoxon-Mann-Whitney U-test. CFU = colony forming units. (DOCX) [file pone.0111627.s002.docx]

**Table S1**

|  | CFU release  *S. aureus* | CFU release  *S. epidermidis* |
| --- | --- | --- |
| MWE Dryswab vs. MWE Σ-Swab | p<0.001 | p<0.001 |
| MWE Dryswab vs. Mast Mastaswab | p<0.01 | p<0.01 |
| MWE Dryswab vs. Sarstedt neutral swab | p<0.001 | p<0.01 |
| MWE Dryswab vs. Copan FLOQSwabs | p<0.001 | p<0.001 |
| MWE Σ-Swab vs. Mast Mastaswab | p<0.001 | p<0.001 |
| MWE Σ-Swab vs. Sarstedt neutral swab | p<0.001 | p<0.001 |
| MWE Σ-Swab vs. Copan FLOQSwabs | p=0.744 | p=0.837 |
| Mast Mastaswab vs. Sarstedt neutral swab | p=0.539 | p=0.539 |
| Mast Mastaswab vs. Copan FLOQSwabs | p<0.001 | p<0.001 |
| Sarstedt neutral swab vs. Copan FLOQSwabs | p<0.001 | p<0.001 |
|  |  |  |
| MWE Dryswab Amies vs. MWE Σ-Swab Amies | p<0.001 | p<0.001 |
| MWE Dryswab Amies vs. Mast Mastaswab Amies | p<0.001 | p<0.001 |
| MWE Dryswab Amies vs. Sarstedt neutral swab Amies | p<0.01 | p<0.001 |
| MWE Dryswab Amies vs. Copan FLOQSwabs Amies | p<0.001 | p<0.001 |
| MWE Σ-Swab Amies vs. Mast Mastaswab Amies | p<0.001 | p<0.001 |
| MWE Σ-Swab Amies vs. Sarstedt neutral swab Amies | p<0.001 | p<0.001 |
| MWE Σ-Swab Amies vs. Copan FLOQSwabs Amies | p=0.325 | p=0.539 |
| Mast Mastaswab Amies vs. Sarstedt neutral swab Amies | p=0.714 | p=0.485 |
| Mast Mastaswab Amies vs. Copan FLOQSwabs Amies | p<0.001 | p<0.001 |
| Sarstedt neutral swab Amies vs. Copan FLOQSwabs Amies | p<0.001 | p<0.001 |
|  |  |  |
| MWE Dryswab Amies vs. MWE Dryswab | p<0.001 | p<0.001 |
| MWE Σ-Swab Amies vs. MWE Σ-Swab | p<0.001 | p<0.001 |
| Mast Mastaswab Amies vs. Mast Mastaswab | p=0.137 | p<0.001 |
| Sarstedt neutral swab Amies vs. Sarstedt neutral swab | p<0.05 | p<0.001 |
| Copan FLOQSwabs Amies vs. Copan FLOQSwabs | p<0.001 | p<0.001 |
