# Supplementary material for: Nasal Screening for MRSA: Different Swabs – Different Results!
Source: PLoS One. 2014 Oct 29;9(10):e111627. doi: 10.1371/journal.pone.0111627 (PMC4213029; doi:10.1371/journal.pone.0111627)
Supplement: Table S2 — Statistics on relative recovery of bacteria. All p values result from nonparametric, two-tailed Wilcoxon-Mann-Whitney U-test. (DOCX) [file pone.0111627.s003.docx]

**Table S2**

|  | *S. aureus* vs.  *S. epidermidis* |
| --- | --- |
| MWE Dryswab | p=0.202 |
| MWE Σ-Swab | p=0.935 |
| Mast Mastaswab | p=0.935 |
| Sarstedt neutral swab | p=0.999 |
| Copan FLOQSwabs | p=0.624 |
|  |  |
| MWE Dryswab Amies | p<0.05 |
| MWE Σ-Swab Amies | p=0.594 |
| Mast Mastaswab Amies | p=0.161 |
| Sarstedt neutral swab Amies | p=0.712 |
| Copan FLOQSwabs Amies | p=0.172 |
