# Supplementary material for: Nasal Screening for MRSA: Different Swabs – Different Results!
Source: PLoS One. 2014 Oct 29;9(10):e111627. doi: 10.1371/journal.pone.0111627 (PMC4213029; doi:10.1371/journal.pone.0111627)
Supplement: Table S3 — Raw data. Results of CFU counting for each experiment are displayed. (DOCX) [file pone.0111627.s004.docx]

**Table S3**

| **Recovered CFU of *S. aureus***  **by direct plating** | | | | | **Recovered CFU of *S. epidermidis***  **by direct plating** | | | | |
| --- | --- | --- | --- | --- | --- | --- | --- | --- | --- |
| **MWE Dryswab** | **MWE Σ-Swab** | **Mast Mastaswab** | **Sarstedt neutral swab** | **Copan FLOQSwabs** | **MWE Dryswab** | **MWE Σ-Swab** | **Mast Mastaswab** | **Sarstedt neutral swab** | **Copan FLOQSwabs** |
| 3 | 9 | 0 | 0 | 9 | 0 | 19 | 0 | 0 | 14 |
| 0 | 0 | 0 | 0 | 4 | 0 | 13 | 0 | 0 | 5 |
| 4 | 8 | 0 | 0 | 11 | 4 | 22 | 1 | 0 | 15 |
| 1 | 2 | 0 | 0 | 30 | 0 | 10 | 0 | 0 | 63 |
| 2 | 1 | 0 | 0 | 13 | 4 | 15 | 0 | 0 | 18 |
| 1 | 22 | 0 | 0 | 47 | 0 | 24 | 0 | 0 | 59 |
| 10 | 37 | 0 | 0 | 8 | 3 | 45 | 0 | 0 | 27 |
| 4 | 10 | 2 | 0 | 36 | 3 | 20 | 0 | 0 | 59 |
| 3 | 15 | 0 | 0 | 32 | 4 | 31 | 0 | 0 | 57 |
| 0 | 19 | 0 | 0 | 21 | 0 | 46 | 1 | 0 | 52 |
| 2 | 28 | 0 | 0 | 10 | 0 | 54 | 0 | 0 | 21 |
| 0 | 40 | 0 | 0 | 18 | 2 | 40 | 0 | 0 | 30 |
| 2 | 20 | 0 | 0 | 15 | 5 | 29 | 0 | 0 | 13 |
| 1 | 7 | 1 | 0 | 7 | 2 | 12 | 0 | 0 | 21 |
| 0 | 19 | 0 | 0 | 5 | 4 | 23 | 0 | 0 | 9 |
|  | | | | | | | | | |
| **Recovered CFU of *S. aureus***  **by elution into Amies medium** | | | | | **Recovered CFU of *S. epidermidis***  **by elution into Amies medium** | | | | |
| **MWE Dryswab** | **MWE Σ-Swab** | **Mast Mastaswab** | **Sarstedt neutral swab** | **Copan FLOQSwabs** | **MWE Dryswab** | **MWE Σ-Swab** | **Mast Mastaswab** | **Sarstedt neutral swab** | **Copan FLOQSwabs** |
| 10 | 200 | 0 | 10 | 150 | 30 | 390 | 10 | 0 | 180 |
| 40 | 180 | 10 | 0 | 160 | 150 | 470 | 10 | 10 | 150 |
| 40 | 250 | 0 | 10 | 160 | 130 | 400 | 10 | 20 | 300 |
| 40 | 110 | 0 | 0 | 250 | 110 | 330 | 20 | 30 | 420 |
| 30 | 170 | 0 | 10 | 230 | 80 | 370 | 10 | 10 | 300 |
| 20 | 210 | 0 | 0 | 140 | 110 | 320 | 10 | 10 | 410 |
| 10 | 270 | 10 | 10 | 210 | 40 | 450 | 20 | 0 | 280 |
| 50 | 150 | 10 | 0 | 290 | 170 | 400 | 10 | 10 | 410 |
| 0 | 120 | 30 | 20 | 90 | 30 | 290 | 40 | 20 | 400 |
| 10 | 140 | 0 | 0 | 240 | 90 | 340 | 10 | 0 | 480 |
| 30 | 190 | 10 | 0 | 350 | 110 | 330 | 30 | 10 | 510 |
| 10 | 270 | 0 | 0 | 240 | 70 | 420 | 10 | 10 | 390 |
| 40 | 80 | 0 | 0 | 200 | 210 | 210 | 20 | 30 | 300 |
| 50 | 170 | 20 | 40 | 260 | 160 | 320 | 20 | 40 | 230 |
| 20 | 210 | 0 | 20 | 170 | 80 | 440 | 0 | 0 | 370 |
