# Supplementary material for: Nasal Screening for MRSA: Different Swabs – Different Results!
Source: PLoS One. 2014 Oct 29;9(10):e111627. doi: 10.1371/journal.pone.0111627 (PMC4213029; doi:10.1371/journal.pone.0111627)
Supplement: Table S4 — Detection limits of swabs with low sensitivities. The minimal bacterial quantities, necessary to achieve positive results after direct plating of the swabs, are displayed for Mast Mastaswab and Sarstedt neutral swab. (DOCX) [file pone.0111627.s005.docx]

**Table S4**

| **Mast Mastaswab** | | | | **Sarstedt neutral swab** | | | |
| --- | --- | --- | --- | --- | --- | --- | --- |
| **Inoculation dose**  ***S. aureus* / *S. epidermidis*** | | | | **Inoculation dose**  ***S. aureus* / *S. epidermidis*** | | | |
| **5x10^2^ / 1x10^3^** | **6x10^2^ / 1.2x10^3^** | **7x10^2^ / 1.4x10^3^** | **8x10^2^ / 1.6x10^3^** | **5x10^2^ / 1x10^3^** | **6x10^2^ / 1.2x10^3^** | **7x10^2^ / 1.4x10^3^** | **8x10^2^ / 1.6x10^3^** |
| **Recovered CFU**  ***S. aureus* / *S. epidermidis*** | | | | **Recovered CFU**  ***S. aureus* / *S. epidermidis*** | | | |
| 0 / 1 | 2 / 0 | 1 / 0 | 1 / 1 | 0 / 0 | 0 / 1 | 1 / 3 | 3 / 2 |
| 2 / 2 | 0 / 0 | 0 / 0 | 2 / 3 | 2 / 1 | 0 / 0 | 0 / 1 | 1 / 5 |
| 1 / 0 | 0 / 1 | 2 / 2 | 0 / 4 | 0 / 0 | 0 / 0 | 1 / 1 | 2 / 2 |
| 0 / 1 | 0 / 0 | 0 / 1 | 1 / 2 | 0 / 5 | 1 / 2 | 3 / 2 | 1 / 1 |
| 0 / 0 | 3 / 1 | 1 / 1 | 2 / 1 | 1 / 1 | 2 / 0 | 1 / 0 | 1 / 2 |
| 0 / 0 | 1 / 1 | 0 / 0 | 1 / 3 | 0 / 0 | 0 / 1 | 1 / 3 | 4 / 7 |
| 1 / 2 | 0 / 1 | 1 / 3 | 4 / 3 | 0 / 2 | 1 / 0 | 0 / 1 | 2 / 3 |
| 1 / 0 | 0 / 0 | 0 / 1 | 1 / 5 | 2 / 0 | 0 / 0 | 2 / 0 | 1 / 4 |
| 0 / 0 | 2 / 0 | 1 / 0 | 1 / 1 | 0 / 1 | 2 / 0 | 1 / 2 | 2 / 2 |
| 0 / 0 | 0 / 2 | 2 / 1 | 2 / 2 | 0 / 0 | 0 / 3 | 1 / 1 | 3 / 2 |
